# Water-Soluble Iridium(III) Complexes Containing Tetraethylene-Glycol-Derivatized Bipyridine Ligands for Electrogenerated Chemiluminescence Detection

**DOI:** 10.3389/fchem.2020.583631

**Published:** 2020-10-15

**Authors:** Ben Newman, Lifen Chen, Luke C. Henderson, Egan H. Doeven, Paul S. Francis, David J. Hayne

**Affiliations:** ^1^School of Life and Environmental Sciences, Faculty of Science, Engineering and Built Environment, Deakin University, Geelong, VIC, Australia; ^2^Institute for Frontier Materials, Deakin University, Geelong, VIC, Australia; ^3^Center for Regional and Rural Futures, Faculty of Science, Engineering and Built Environment, Deakin University, Geelong, VIC, Australia

**Keywords:** electrogenerated chemiluminescence, electrochemiluminescence, transition metal complexes, iridium(III) complexes, labeling

## Abstract

Four cationic heteroleptic iridium(III) complexes containing a 2,2′-bipyridine (bpy) ligand with one or two tetraethylene glycol (TEG) groups attached in the 4 or 4,4′ positions were synthesized to create new water-soluble electrogenerated chemiluminescence (ECL) luminophores bearing a convenient point of attachment for the development of ECL-labels. The novel TEG-derivatized bipyridines were incorporated into [Ir(C^∧^N)_2_(R-bpy-R′)]Cl complexes, where C^∧^N = 2-phenylpyridine anion (ppy) or 2-phenylbenzo[d]thiazole anion (bt), through reaction with commercially available ([Ir(C^∧^N)_2_(μ-Cl)]_2_ dimers. The novel [Ir(C^∧^N)_2_(Me-bpy-TEG)]Cl and [Ir(C^∧^N)_2_(TEG-bpy-TEG)]Cl complexes in aqueous solution largely retained the redox potentials and emission spectra of the parent [Ir(C^∧^N)_2_(Me-bpy-Me)]PF_6_ (where Me-bpy-Me = 4,4′methyl-2,2′-bipyridine) luminophores in acetonitrile, and exhibited ECL intensities similar to those of [Ru(bpy)_3_]^2+^ and the analogous [Ir(C^∧^N)_2_(pt-TEG]Cl complexes (where pt-TEG = 1-(TEG)-4-(2-pyridyl)-1,2,3-triazole). These complexes can be readily adapted for bioconjugation and considering the spectral distributions of [Ir(ppy)_2_(Me-bpy-TEG)]^+^ and [Ir(ppy)_2_(pt-TEG)]^+^, show a viable strategy to create ECL-labels with different emission colors from the same commercial [Ir(ppy)_2_(μ-Cl)]_2_ precursor.

## Introduction

Electrogenerated chemiluminescence (ECL) is the process whereby electrochemically oxidized and reduced species undergo subsequent electron transfer reactions to produce electronically excited products that emit light (Bard, 2004; Miao, 2008). To date, the wide use of ECL across various fields has predominantly focused on ruthenium(II) complexes [particularly [Ru(bpy)_3_]^2+^, where bpy = 2,2′-bipyridine] as the luminophores (Liu et al., 2015; Muzyka et al., 2017; Qi and Zhang, 2020). These complexes are highly soluble in buffered aqueous solution and generally produce ECL in the red/orange region of the electromagnetic spectrum. Cyclometalated iridium(III) complexes have attracted enormous interest as alternative ECL luminophores to the conventional ruthenium(II) complexes due to their high luminescence efficiencies and wide range of emission colors (Kapturkiewicz, 2016; Fiorani et al., 2018; Haghighatbin et al., 2018), which not only enables the emission to be shifted into the region where commonly used photomultiplier tubes are most sensitive (Barbante et al., 2014a; Kerr et al., 2015), but also creates new opportunities for tunable light-emitting devices (Moon et al., 2014; Kwon and Myoung, 2019; Soulsby et al., 2019; Cho et al., 2020) and simultaneous detection of spectrally distinct species (Doeven et al., 2012, 2013, 2015b; Barbante et al., 2014b; Soulsby et al., 2018).

Numerous cyclometalated iridium(III) complexes have been synthesized and many have shown impressive annihilation and/or co-reactant ECL intensities in organic media (Bruce and Richter, 2002; Kapturkiewicz et al., 2004; Kim et al., 2005). For example, we recently re-examined a promising series of heteroleptic iridium(III) complexes containing an acetylacetonate anion (acac) ligand, with several exhibiting much greater ECL intensities than [Ru(bpy)_3_]^2+^ (with tri-*n*-propylamine (TPrA) co-reactant in acetonitrile solution), although the relative intensities were highly dependent on reaction conditions (Chen et al., 2017).

**Figure 1:**
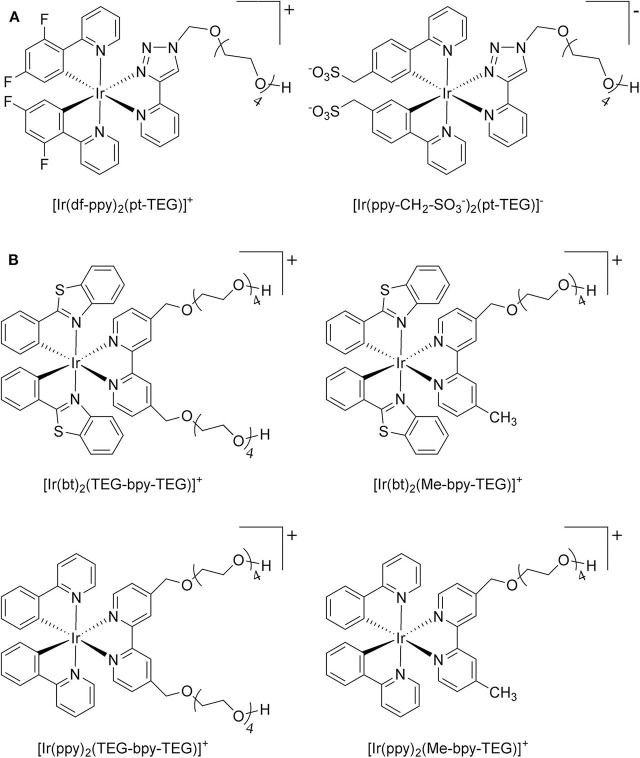
**(A)** [Ir(C^∧^N)_2_(pt-TEG)]^+^ complexes (Kerr et al., 2015) and **(B)** the novel iridium(III) complexes containing a 2,2′-bipyridine ligand with one or two tetraethylene glycol (TEG) groups.

Nevertheless, very few of the iridium(III) complexes examined as ECL luminophores to date are soluble in the aqueous conditions in which most ECL assays are performed (Fernandez-Hernandez et al., 2016; Zhou et al., 2017). As previously reported, the solubility can be improved by incorporating polar functional groups such as sulfonates (Kiran et al., 2009; Jia et al., 2012) or saccharides (Li et al., 2011a,b) on one or more ligands of the complex. Li et al. (2011b), for example, reported intense ECL from a water-soluble bis-cyclometalated iridium(III) complex incorporating a bpy ligand appended with two sugar moieties. Similarly, we utilized bathophenanthroline-disulfonate (BPS) as an ancillary ligand to increase the solubility of the complexes in aqueous solution (Kiran et al., 2009; Zammit et al., 2011; Truong et al., 2014). In most cases, however, the dissolution of the complexes at relatively high concentrations often required the addition of some acetonitrile to the aqueous solution, and these approaches do not provide a convenient means to incorporate the luminophores into ECL labels. We recently examined the ECL of several water soluble [Ir(C^∧^N)_2_(pt)]Cl complexes (where C^∧^N = 2-phenylpyridine anion (ppy) or 2-(2,4-difluorophenyl)pyridine anion (df-ppy), and pt = 4-(2-pyridyl)-1,2,3-triazole) with either a tetraethylene glycol (TEG) or benzyl group attached to the triazole and/or methanesulfonate substituents on the ppy/df-ppy ligands (Doeven et al., 2015a; Kerr et al., 2015). Although the TEG and methanesulfonate groups improved the solubility of the complexes in water, the complexes with the pt-TEG ligand (Figure 1A) gave greater co-reactant ECL intensities with TPrA and provide a convenient point of attachment of functional groups for bioconjugation (Connell et al., 2015; Chen et al., 2019) for the development of iridium(III) complex ECL labels.

Herein, we prepare four novel [Ir(C^∧^N)_2_(N^∧^N)]Cl complexes (Figure 1B), where N^∧^N is bpy with either one or two TEG groups attached in the 4 and 4′ positions (referred to hereafter as Me-bpy-TEG and TEG-bpy-TEG). Through the introduction of the TEG group(s) onto the commonly used bpy ligand, iridium(III) complexes previously studied in organic solvents can be examined in buffered aqueous solution. We incorporate the Me-bpy-TEG and TEG-bpy-TEG ligands into heteroleptic iridium(III) complexes with ppy or 2-phenylbenzo[d]thiazole anion (bt) ligands by reacting the bipyridine derivatives with commercially available ([Ir(C^∧^N)_2_(μ-Cl)]_2_ dimers. We evaluate the influence of the TEG group(s) on the parent luminophore by comparing their spectroscopic and electrochemical properties with the corresponding [Ir(C^∧^N)_2_(Me-bpy-Me)]^+^ complexes, and compare their co-reactant ECL intensities to the analogous water-soluble [Ir(C^∧^N)_2_(pt-TEG)]^+^ complexes and [Ru(bpy)_3_]^2+^.

## Materials and Methods

### Chemicals and General Details

Tris(2,2′-bipyridine)ruthenium(II) hexafluorophosphate ([Ru(bpy)_3_](PF_6_)_2_) and tetrabutylammonium hexafluorophosphate (TBAPF_6_; electrochemical grade) were purchased from Sigma-Aldrich (NSW, Australia). Bis(cyclopentadienyl)iron (ferrocene; Fc) and tris(2,2′-bipyridine)ruthenium(II) dichloride hexahydrate ([Ru(bpy)_3_]Cl_2_.6H_2_O) were purchased from Strem Chemicals (MA, USA). The iridium(III) dimer precursors were purchased from SunaTech (China). Reagents and solvents were purchased from various commercial sources and used without further purification. NMR spectra were acquired on a Bruker Biospin AV400 spectrometer or a Bruker Biospin AV500 spectrometer. ^1^H NMR spectra were acquired at 400 or 500 MHz, and ^13^C{^1^H} NMR spectra were acquired at 100 or 126 MHz. All NMR spectra were recorded at 298 K. Chemical shifts were referenced to residual solvent peaks and are quoted in parts per million (ppm), relative to tetramethylsilane (Si(CH_3_)_4_).

### Synthesis

#### 1,1,1-triphenyl-2,5,8,11-tetraoxatridecan-13-ol


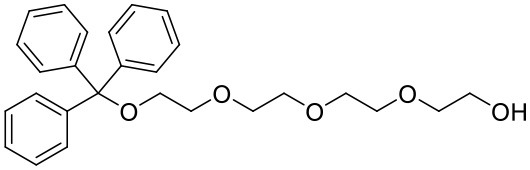


Trityl chloride (4.85 g, 17.4 mmol) in dichloromethane (80 mL) was added dropwise to a mixture of tetraethylene glycol (33.1 g, 170.6 mmol) and triethylamine (8 mL, 57.4 mmol) in dichloromethane (200 mL). After the addition was complete, the reaction mixture was stirred for 2 days at ambient temperature. The mixture was washed with saturated sodium carbonate (200 mL), dH_2_O (3 × 200 mL) then brine (200 mL) before being dried (MgSO_4_). The solvent was removed under reduced pressure to afford a yellow oil (6.95 g, 15.9 mmol, 91%). ^1^H NMR (500 MHz; CD_3_CN): δ 7.47–7.44 (m, 6H), 7.34–7.31 (m, 6H), 7.28–7.24 (m, 3H), 3.62–3.55 (m, 12H), 3.48–3.46 (m, 2H), 3.14 (dd, J = 5.4, 4.3, 2H). ^13^C{^1^H} NMR (101 MHz, CD_3_CN): δ 145.27, 129.55, 128.82, 128.05, 87.35, 73.30, 71.43, 71.27, 71.24, 71.12, 71.09, 64.39, 61.95.

#### 1,1,1-triphenyl-2,5,8,11-tetraoxatridecan-13-yl 4-methylbenzenesulfonate


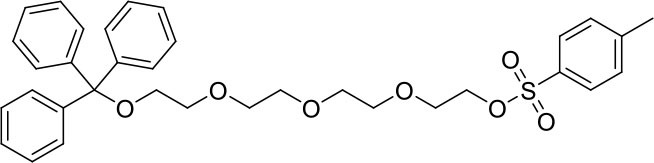


A mixture of 1,1,1-triphenyl-2,5,8,11-tetraoxatridecan-13-ol (3.51 g, 8.1 mmol) in tetrahydrofuran (100 mL) was cooled to 0°C. Sodium hydroxide (8 M, 25 mL) was added followed by dropwise addition of tosylchloride (1.85 g, 9.7 mmol) in tetrahydrofuran (80 mL). The reaction was left to warm to ambient temperature and stirred overnight. Brine (100 mL) was added to the mixture which was then extracted with dichloromethane (3 × 75 mL). The combined organic extracts were dried (MgSO_4_) and the solvent was removed under reduced pressure to afford a yellow oil (4.58 g, 7.6 mmol, 91%). ^1^H NMR (500 MHz; CD_3_CN): δ 7.78–7.75 (m, 2H), 7.46–7.44 (m, 6H), 7.42–7.40 (m, 2H), 7.33–7.30 (m, 6H), 7.27–7.23 (m, 3H), 4.08–4.06 (m, 2H), 3.61–3.54 (m, 8H), 3.53–3.51 (m, 2H), 3.49–3.47 (m, 2H), 3.14–3.12 (m, 2H), 2.41 (s, 3H). ^13^C{^1^H} NMR (101 MHz, CD_3_CN): δ 146.34, 145.26, 133.79, 131.00, 129.54, 128.82, 128.77, 128.05, 87.34, 71.42, 71.25, 71.20, 71.11, 70.94, 69.17, 64.39, 21.65.

#### L^1^Trt


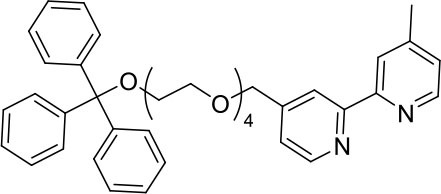


A mixture of 4-(hydroxymethyl)-4′-methyl-2,2′-bipyridine (458 mg, 2.3 mmol) and sodium hydride (60% oil dispersion, 190 mg, 4.8 mmol) was heated at reflux in tetrahydrofuran (dry, 50 mL). After 1.5 h, 1,1,1-triphenyl-2,5,8,11-tetraoxatridecan-13-yl 4-methylbenzenesulfonate (1,662 mg, 2.8 mmol) was added and the reaction mixture was stirred at reflux for 20 h. The reaction mixture was cooled to ambient temperature before methanol was added to quench excess sodium hydride. A solid was observed and remove by filtration then the solvent was removed from the filtrate under reduced pressure. The residue was purified by flash chromatography (SiO_2_, 0 → 50% ethyl acetate in dichloromethane) to afford the product as a viscous yellow oil after removal of the solvent under reduced pressure (940 mg, 1.5 mmol, 65%) ^1^H NMR (400 MHz; CD_3_CN): δ 8.57 (d, J = 4.9, 1H), 8.49 (d, J = 5.0, 1H), 8.36 (s, 1H), 8.26 (s, 1H), 7.44 (m, 6H), 7.31 (m, 7H), 7.30 (m, 4H), 4.62 (s, 2H), 3.61 (m, 14H), 3.11 (t, J = 4.8, 2H), 2.42 (s, 3H). ^13^C{^1^H} NMR (101 MHz, CD_3_CN): δ 156.97, 156.57, 150.10, 150.02, 149.95, 149.27, 145.20, 129.47, 128.74, 127.97, 125.74, 122.77, 122.33, 119.58, 87.26, 71.99, 71.38, 71.23, 71.21, 71.07, 71.04, 71.00, 64.32, 21.20.

#### L^1^ (Me-bpy-TEG)


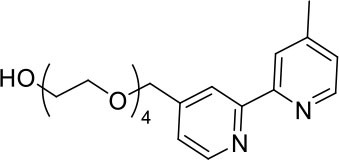


A mixture of **L**^**1**^**Trt** (940 mg, 1.52 mmol) and trifluoroacetic acid (2 mL) was set stirring in dichloromethane (20 mL) at ambient temperature. After 1 h the mixture was extracted with 1 M hydrochloric acid (3 × 30 mL) and the combined aqueous extracts were adjusted to pH 9 by careful addition of solid potassium carbonate. The aqueous phase was then extracted with ethyl acetate (3 × 30 mL) and the combined organic extracts were dried and the solvent removed under reduced pressure to afford a yellow oil (229 mg, 0.61 mmol, 40%). ^1^H NMR (400 MHz, CD_3_CN): δ 8.61 (dd, *J* = 5.0, 0.7 Hz, 1H), 8.51 (dd, *J* = 5.0, 0.5 Hz, 1H), 8.35 (dd, *J* = 1.6, 0.8 Hz, 1H), 8.28–8.24 (m, 1H), 7.34 (ddd, *J* = 7.3, 4.0, 3.2 Hz, 1H), 7.26–7.21 (m, 1H), 4.66 (s, 2H), 3.70–3.51 (m, 14H), 3.49–3.44 (m, 2H), 2.46 (d, *J* = 20.2 Hz, 3H). ^13^C{^1^H} NMR (101 MHz, CD_3_CN): δ 157.01, 156.57, 150.19, 149.93, 149.80, 149.43, 128.65, 125.78, 122.95, 122.45, 119.71, 73.10, 72.04, 70.97, 70.91, 70.88, 70.84, 70.74, 61.69, 21.20.

#### 4,4′-bis(ethoxycarbonyl)-2,2′-bipyridine


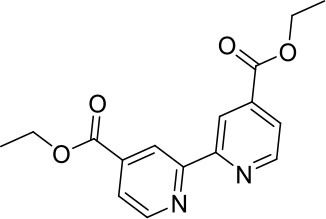


A mixture of 2,2′-bipyridine-4,4′-dicarboxylic acid (5.08 g, 20.8 mmol) and sulfuric acid (10 mL) was heated at reflux in ethanol (200 mL). After 24 h the reaction mixture was cooled to ambient temperature then poured into ice water (100 mL) and adjusted to pH 7 by addition of solid potassium carbonate. The solvent volume was lessened under reduced pressure, and the mixture was extracted with dichloromethane (150 mL). The organic phase was washed with dH_2_O (3 × 100 mL) then brine (100 mL) before drying (MgSO_4_) and removal of the solvent under reduced pressure to afford a colorless solid (4.85 g, 16.1 mmol, 77%). ^1^H NMR (400 MHz, CDCl_3_): δ 8.95 (s, 1H), 8.86 (s, 1H), 7.91 (d, J = 4.2 Hz, 1H), 4.46 (q, J = 7.1 Hz, 2H), 1.44 (t, J = 7.1 Hz, 3H). ^13^C{^1^H} NMR (101 MHz, CDCl_3_): δ 165.24, 156.46, 150.15, 139.22, 123.47, 120.79, 62.09, 14.42.

#### 4,4′-bis(hydroxymethyl)-2,2′-bipyridine


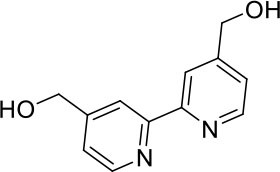


A mixture of 4,4′-bis(ethoxycarbonyl)-2,2′-bipyridine (2.80 g, 9.3 mmol) in ethanol (150 mL) was set stirring and sodium borohydride (3.83 g, 85 mmol) was added portion-wise. After stirring for 16 h at ambient temperature, excess sodium borohydride was quenched by addition of an aqueous solution of saturated ammonium chloride. The reaction mixture was filtered to remove solids, and the solvent volume was lessened under reduced pressure. The mixture was washed with ethyl acetate (6 × 100 mL) and the combined organic extracts were washed with brine. The organic phase was dried (MgSO_4_) and the solvent removed under reduced pressure to afford a colorless solid (1.51 g, 7.0 mmol, 75%). ^1^H NMR (400 MHz, DMSO): δ 8.60 (d, *J* = 5.0 Hz, 1H), 8.39 (s, 1H), 7.37 (d, *J* = 4.9 Hz, 1H), 5.52 (t, *J* = 5.8 Hz, 1H), 4.63 (d, *J* = 5.7 Hz, 2H). ^13^C{^1^H} NMR (101 MHz, d_6_-DMSO): δ 155.21, 152.83, 148.96, 121.40, 117.75, 61.71.

#### L^2^Trt_2_


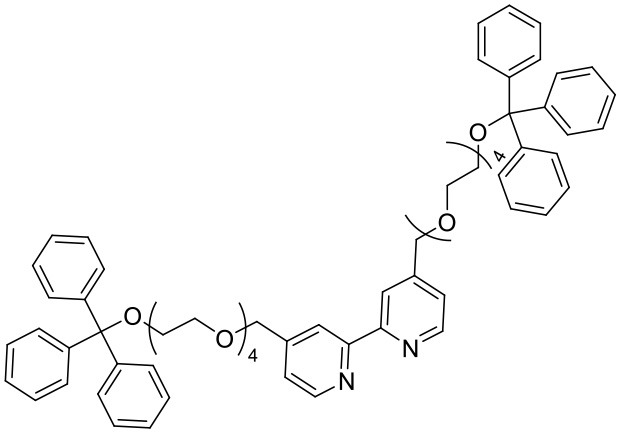


A mixture of 4,4′-bis(hydroxymethyl)-2,2′-bipyridine (254 mg, 1.2 mmol) and sodium hydride (60% oil dispersion, 70 mg, 1.8 mmol) was heated at reflux in tetrahydrofuran (dry, 50 mL). After 1.5 h, 1,1,1-triphenyl-2,5,8,11-tetraoxatridecan-13-yl 4-methylbenzenesulfonate (1,693 mg, 2.9 mmol) was added and the reaction mixture was stirred at reflux for 20 h. The reaction mixture was cooled to ambient temperature before methanol was added to quench residual sodium hydride. The reaction mixture was filtered to remove any solids and the solvent was removed under reduced pressure. The resultant residue was taken up in ethyl acetate (100 mL) and washed with dH_2_O (3 × 100 mL) then brine (100 mL). The organic phase was dried (MgSO_4_) and the solvent was removed under reduced pressure, then the residue was purified by column chromatography (SiO_2_, 0 → 10% methanol in ethyl acetate) to afford a yellow oil (939 mg, 0.89 mmol, 74%). ^1^H NMR (400 MHz, CD_3_CN): δ 8.57 (d, 2H), 8.37 (d, 2H), 7.49–7.40 (m, 12H), 7.32 (s, 2H), 7.32–7.27 (m, 12H), 7.26–7.20 (m, 6H), 4.06 (q, 2H), 3.68–3.49 (m, 30H), 3.16–3.06 (m, 4H). ^13^C{^1^H} NMR (126 MHz, CD_3_CN): δ 156.83, 150.16, 145.23, 129.51, 128.79, 128.01, 122.92, 119.60, 87.29, 70.99, 64.36, 60.95.

#### General Method for Synthesis of Iridium Complexes

The chloro-bridged iridium dimer and almost two molar equivalents of ligand were added to a flask. A solvent mixture of dichloromethane and methanol (1:1 v/v) was added, and the mixture was sparged with N_2_ for 20 min then sealed, shielded from light and heated at 50°C for 20 h. Any solid that remained in the reaction mixture was removed by centrifuge and the supernatant was filtered through filter aid (Celite). The solvent was removed under reduced pressure, the residue was taken up in a minimum of dichloromethane and a precipitate was formed after addition of diethyl ether. The precipitate was isolated by centrifuge and washed with diethyl ether (× 3) then dried *in vacuo*.

#### [Ir(bt)_2_(Me-bpy-TEG)]Cl


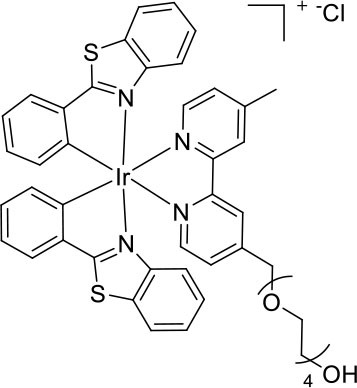


The dimer [Ir(bt)_2_(μ-Cl)]_2_ (239 mg, 0.18 mmol) and **L**^**1**^ (132 mg, 0.35 mmol) were reacted according to the general method. The product was isolated as an orange powder (250 mg, 0.24 mmol, 69%). ESI-MS (positive ion). Calcd for C_46_H_44_IrN_4_O_5_${\text{S}}_{2}^{+}$ ([M]^+^): *m/z* 989.238. Found *m/z* 989.2381.^1^H NMR (500 MHz, CD_3_CN): δ 8.52 (s, 1H), 8.44 (s, 1H), 8.03 (t, J = 7.1 Hz, 3H), 7.92 (dd, J = 8.5, 7.0 Hz, 3H), 7.52 (d, J = 5.6 Hz, 1H), 7.38 (t, J = 7.4 Hz, 3H), 7.12 (dt, J = 21.1, 7.6 Hz, 4H), 6.87 (t, J = 7.5 Hz, 2H), 6.40 (dd, J = 7.5, 4.9 Hz, 2H), 6.24 (dd, J = 8.4, 4.2 Hz, 2H), 4.77 (s, 2H), 3.65 (ddd, J = 8.4, 6.4, 3.5 Hz, 4H), 3.55–3.44 (m, 10H), 3.39–3.35 (m, 2H), 2.54 (s, 3H). ^13^C{^1^H} NMR (126 MHz, CD_3_CN): δ 182.47, 182.44, 157.42, 156.99, 154.26, 153.66, 151.66, 151.64, 151.57, 151.10, 150.13, 150.11, 141.47, 141.43, 134.19, 134.15, 132.84, 132.67, 132.63, 130.38, 129.05, 129.00, 127.75, 127.20, 127.04, 127.02, 126.39, 124.94, 124.12, 122.93, 73.28, 71.35, 71.15, 71.11, 71.07, 71.05, 70.99, 70.96, 61.76, 21.49.

#### [Ir(ppy)_2_(Me-bpy-TEG)]Cl


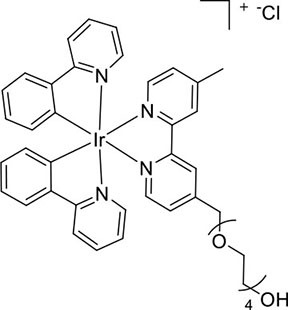


The dimer [Ir(ppy)_2_(μ-Cl)]_2_ (193 mg, 0.18 mmol) and **L**^**1**^ (128 mg, 0.34 mmol) were reacted according to the general method. The product was isolated as a yellow powder (243 mg, 0.27 mmol, 79%). ESI-MS (positive ion). Calcd for C_42_H_44_IrN_4_${\text{O}}_{5}^{+}$ ([M]^+^): *m/z* 877.294. Found *m/z* 877.2937. ^1^H NMR (500 MHz, CD_3_CN): δ 8.58 (s, 1H), 8.50 (s, 1H), 8.06 (d, J = 8.1 Hz, 2H), 7.89 (d, J = 5.6 Hz, 1H), 7.86–7.76 (m, J = 11.7, 6.1 Hz, 5H), 7.61 (dd, J = 12.2, 5.7 Hz, 2H), 7.45 (d, J = 5.6 Hz, 1H), 7.31 (d, J = 5.6 Hz, 1H), 7.03 (t, J = 7.0 Hz, 4H), 6.90 (t, J = 7.4 Hz, 2H), 6.27 (dd, J = 7.5, 4.4 Hz, 2H), 4.75 (s, 2H), 3.72–3.63 (m, 4H), 3.59–3.45 (m, J = 29.1, 5.3 Hz, 10H), 3.41 (t, J = 4.9 Hz, 2H), 2.53 (s, 3H). ^13^C{^1^H} NMR (126 MHz, CD_3_CN): δ 168.44, 168.41, 156.82, 156.39, 153.53, 152.98, 151.65, 151.61, 151.18, 150.73, 150.13, 150.05, 145.07, 145.03, 139.43, 132.53, 132.50, 131.29, 131.28, 129.98, 126.87, 126.52, 125.83, 125.81, 124.44, 124.43, 123.38, 123.37, 123.17, 120.80, 120.77, 73.29, 71.34, 71.16, 71.14, 71.12, 71.05, 70.99, 70.96, 61.74, 21.43.

#### [Ir(ppy)_2_(L^2^Trt_2_)]Cl


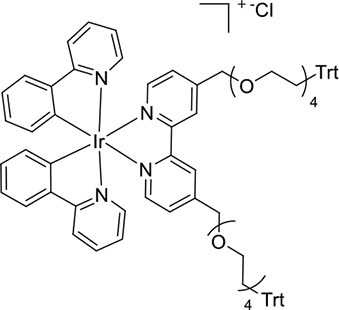


The dimer [Ir(ppy)_2_(μ-Cl)]_2_ (71.7 mg, 0.067 mmol) and **L**^**2**^Trt_2_ (118 mg, 0.112 mmol) were reacted according to the general method. The product was isolated as a yellow powder (106 mg, 0.067 mmol, 60%). ^1^H NMR (400 MHz, CD_3_CN): δ 8.56–8.42 (m, 4H), 8.03 (dd, 6H), 7.97–7.73 (m, 8H), 7.60–7.52 (m, 12H), 7.41–7.18 (m, 12H), 7.08–6.85 (m, 6H), 6.27 (t, 4H), 4.71 (dd, 4H), 3.58 (dt, 32H). ^13^C{^1^H} NMR (126 MHz, CD_3_CN): δ 151.21, 145.19, 139.41, 128.03, 126.98, 125.84, 124.39, 123.44, 120.81, 73.28, 70.71, 64.22, 61.86.

#### [Ir(bt)_2_(L^2^Trt_2_)]Cl


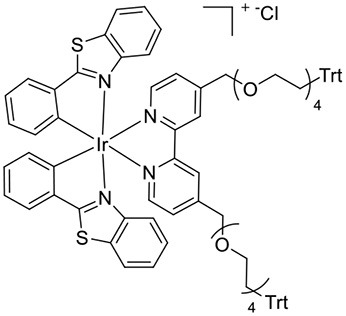


The dimer [Ir(bt)_2_(μ-Cl)]_2_ (111 mg, 0.086 mmol) and **L**^**2**^Trt_2_ (162 mg, 0.15 mmol) were reacted as per the general method. The product was isolated as an orange powder (168 mg, 0.099 mmol, 66%). ^1^H NMR (400 MHz, CD_3_CN) δ 8.48 (s, 2H), 8.04 (dd, 4H), 7.92 (d, 2H), 7.55 (d, 2H), 7.39 (t, 2H), 7.13 (dd, 4H), 6.89 (t, 2H), 6.40 (d, 2H), 6.24 (d, 2H), 4.78 (s, 4H), 3.65 (dd, 8H), 3.58–3.33 (m, 26H). ^13^C{^1^H} NMR (126 MHz, CD_3_CN) δ 182.04, 156.81, 153.84, 151.16, 149.68, 141.01, 133.74, 132.44, 132.22, 128.62, 127.34, 126.86, 126.61, 124.50, 123.74, 122.64, 72.81, 70.43, 61.41.

#### General Procedure for Trityl Deprotection of [Ir(C^∧^N)_2_(L^2^Trt_2_)]Cl Complexes

The trityl protected complex was set stirring in methanol (15 mL) and the mixture was cooled to 0°C before acetyl chloride (0.4 mL) was added. The mixture was then stirred for a total of 10 h at ambient temperature. The solvent was removed under reduced pressure, and the residue was taken up in a minimum of dichloromethane. A precipitate was formed upon addition of diethyl ether and isolated by centrifugation, washed with diethyl ether (× 3) then dried *in vacuo*.

#### [Ir(ppy)_2_(TEG-bpy-TEG)]Cl


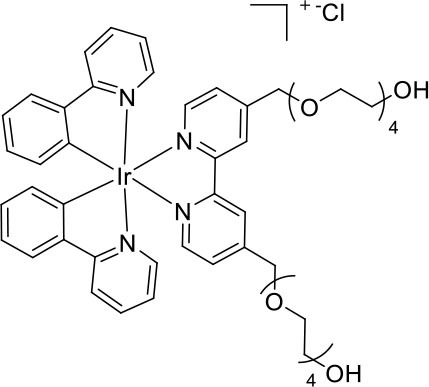


The complex [Ir(ppy)_2_(L^2^Trt_2_)]Cl (90 mg, 0.057 mmol) was reacted according to the general procedure for trityl deprotection to afford a yellow solid (51 mg, 0.046 mmol, 81%). ESI-MS (positive ion). Calcd for C_50_H_60_IrN_4_${\text{O}}_{10}^{+}$ ([M]^+^): *m/z* 1069.394. Found *m/z* 1069.3932. ^1^H NMR (400 MHz, CD_3_CN): δ 8.52 (s, 2H), 8.03 (dd, 2H), 7.97–7.77 (m, 6H), 7.62 (d, 2H), 7.48 (d, 2H), 7.04 (t, 4H), 6.91 (t, 2H), 6.28 (d, 2H), 4.76 (s, 4H), 3.76–3.38 (m, 32H). ^13^C{^1^H} NMR (126 MHz, CD_3_CN): δ 168.53, 156.59, 153.44, 151.29, 150.18, 145.29, 139.47, 132.53, 131.13, 126.99, 125.84, 124.46, 123.36, 120.81, 73.28, 70.85, 61.86.

#### [Ir(bt)_2_(TEG-bpy-TEG)]Cl


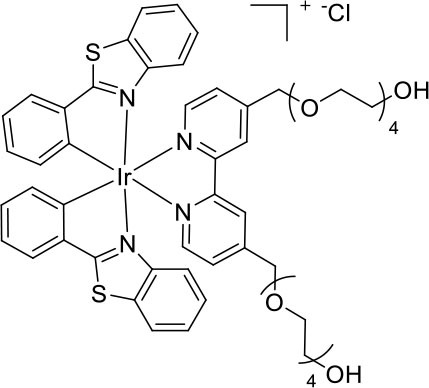


The complex [Ir(bt)2(bpy-(TEG-Trt)_2_)]Cl (101 mg, 0.059 mmol) was reacted according to the general procedure for trityl deprotection to afford an orange solid (53 mg, 0.043 mmol, 73%). ESI-MS (positive ion). Calcd for C_54_H_60_IrN_4_O_10_${\text{S}}_{2}^{+}$ ([M]^+^): *m/z* 1181.338. Found *m/z* 1181.3377. ^1^H NMR (400 MHz, CD_3_CN): δ 8.47 (s, 2H), 8.16–8.00 (m, 4H), 7.92 (d, 2H), 7.56 (t, 2H), 7.39 (t, 1H), 7.12 (dd, 4H), 6.88 (t, 2H), 6.40 (d, 2H), 6.24 (d, 2H), 4.76 (d, 4H), 3.65 (dd, 9H), 3.59–3.30 (m, 25H). ^13^C{^1^H} NMR (126 MHz, CD_3_CN): δ 182.04, 156.81, 153.84, 151.16, 149.68, 141.01, 133.74, 132.33, 128.62, 127.34, 126.74, 124.50, 123.74, 122.64, 72.81, 70.87, 61.41.

### Absorbance and Emission Spectra

Absorbance spectra were obtained using a Cary 300 Bio UV/Vis spectrophotometer (Agilent, USA). Conditions: Double beam mode; 2 nm SBW; 1 nm data interval; 600 nm/min scan rate; 0.1 s averaging time. Quartz cuvettes with a path length of 1 cm were used for all measurements. All room temperature photoluminescence spectra were collected using a Cary Eclipse spectrofluorometer. Conditions: 5 nm band pass; 1 nm data interval; 600 nm/min scan rate; 0.1 s averaging time; 250–395 nm excitation filter; 360–1,100 nm emission filter; PMT: 600 V; final spectrum is an average of 10 scans for [Ir(bt)_2_(Me-bpy-TEG)]^+^ and [Ir(bt)_2_(TEG-bpy-TEG)]^+^, and 50 for [Ir(ppy)_2_(Me-bpy-TEG)]^+^ and [Ir(ppy)_2_(TEG-bpy-TEG)]^+^ (CAT mode). Quartz cuvettes with a path length of 1 cm were used for all measurements. Low temperature spectra were obtained using an OptistatDN Variable Temperature Liquid Nitrogen Cryostat (Oxford Instruments) with custom-made quartz sample holder, placed within the Eclipse sample chamber. Low temperature spectra were collected at 85 K to avoid damage to the spectroscopic cuvettes near 77 K. No difference was observed in the λ_max_ at 77 K and 85 K for complexes such as [Ru(bpy)_3_]^2+^ under these conditions (Soulsby et al., 2018).

In both the low temperature and room temperature data, there is a wavelength dependence of the detector response. To account for this, a correction factor (established using a quartz-halogen tungsten lamp of standard spectral irradiance) was applied to both room temperature and low temperature emission spectra. All room temperature experiments were performed with deionized water or acetonitrile, and all low temperature experiments performed in an ethanol:methanol (4:1) glass.

### Electrochemistry and ECL

An Autolab PGSTAT204 or PGSTAT128N potentiostat (Metrohm Autolab B.V., Netherlands) was used to perform cyclic voltammetry, squarewave voltammetry (0.005 V step, 0.02 V amplitude, 25 Hz), and chronoamperometry (CA). The system comprised of a flat-bottomed glass electrochemical cell with a Teflon custom-built lid designed for a three-electrode system. The electrodes were a glassy carbon working (CH instruments), Pt wire counter, and either a “leakless” Ag/AgCl reference (Innovative Instruments, FL, USA) or Ag wire pseudo-reference. This configuration positioned the working electrode 2 mm from the bottom of the cell. Experiments were conducted with the electrochemical cell housed in a Faraday cage. All Experiments performed in acetonitrile were referenced to Fc^+/0^ *in situ* (at equimolar concentration to the analyte). The working electrode was polished on a felt pad with 0.05 μm alumina powder prior to use. A small blowtorch was used to polish the platinum electrode prior to use. All solutions were prepared in either deionized water with a 0.1 M phosphate buffer adjusted to pH 7.5, or dry acetonitrile with 0.1 M TBAPF_6_ electrolyte, and were deaerated with nitrogen gas for 5 min. ECL spectra were collected using a QE65pro Ocean Optics CCD *via* optical fiber and collimating lens positioned below the base of the electrochemical cell. Each acquisition was triggered by the potentiostat in conjunction with a HR4000 Break-Out box. Relative ECL intensities were averages of two replicates using the integrated areas under the spectra.

## Results and Discussion

### Synthesis of Iridium(III) Complexes

**Scheme 1:**
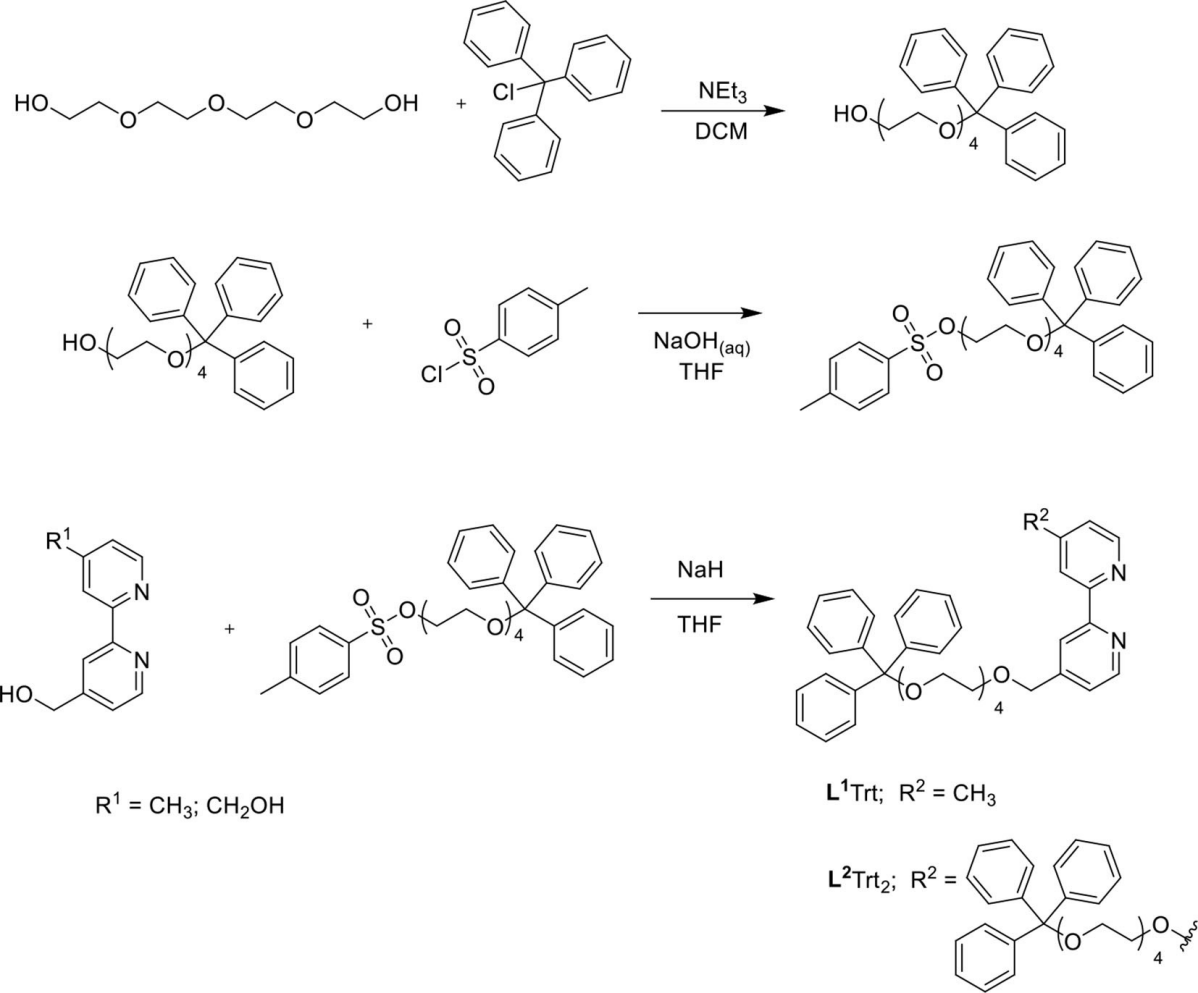
Synthesis of trityl protected bipyridine ligands (L^1^Trt and L^2^Trt_2_).

To prepare the complexes shown in Figure 1B, the chloro-bridged iridium(III) dimers ([Ir(C^∧^N)_2_(μ-Cl)]_2_, where C^∧^N = ppy or bt) were initially reacted with bpy derivatives furnished with either one or two TEG groups (Me-bpy-TEG (**L**^**1**^) and TEG-bpy-TEG (**L**^**2**^)). The bipyridine ligands were prepared using TEG mono-protected with a trityl group in excellent yield (91%) and converted to the corresponding tosyl ester (91%) to afford a suitable leaving group to react with hydroxyl methyl bipyridine derivatives (Scheme 1).

Trifluoroacetic acid was used to deprotect **L**^**1**^Trt followed by acid/base extraction to give **L**^**1**^, but **L**^**2**^ proved difficult to isolate by this method, presumably due to the water solubility of the product. Therefore, **L**^**2**^Trt_2_ was used directly to form iridium(III) dimers and subsequent removal trityl groups, in methanolic hydrochloric acid, gave [Ir(C^∧^N)_2_(TEG-bpy-TEG)]Cl in good to excellent yields. This strategy proved beneficial as the presence of the trityl group allowed the complex to be isolated *via* traditional silica gel chromatography, and then the desired water solubility could be introduced as the final step. Precipitation of complexes from dichloromethane occurred upon addition of diethyl ether allowing isolation by centrifugation. The [Ir(C^∧^N)_2_(Me-bpy-TEG)]Cl and [Ir(C^∧^N)_2_(TEG-bpy-TEG)]Cl complexes were sufficiently soluble for the preparation of aqueous stock solutions at 1 mM.

### UV-Vis Absorption and Photoluminescence Spectra

**Figure 2:**
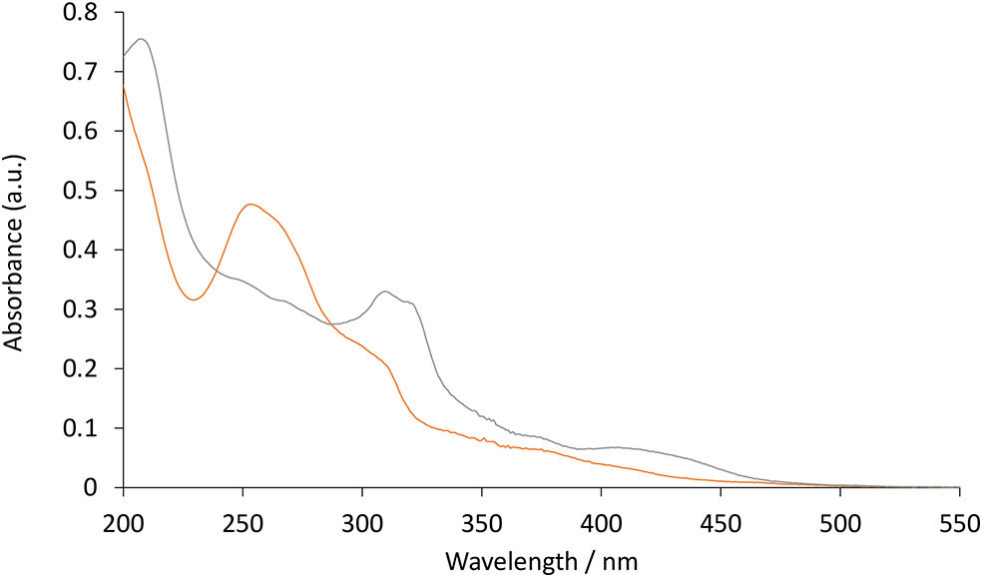
UV-vis absorbance spectra obtained for [Ir(bt)_2_(TEG-bpy-TEG)]^+^ (gray line), and [Ir(ppy)_2_(TEG-bpy-TEG)]^+^ (orange line), at a concentration of 10 μM in water at ambient temperature.

UV-vis absorption spectra of the four novel iridium(III) complexes and [Ru(bpy)_3_]^2+^ were examined at 10 μM in water (e.g., Figure 2) and the peak maxima were compared to the [Ir(C^∧^N)_2_(Me-bpy-Me)]^+^ analogs at the same concentration in acetonitrile (Table 1). The two [Ir(C^∧^N)_2_(TEG-bpy-TEG)]^+^ complexes showed similar absorption spectra to their [Ir(C^∧^N)_2_(Me-bpy-TEG)]^+^ counterparts in water. In general, complexes with the ppy ligands exhibited strong π → π^*^ LC transitions (λ = 240–300 nm), while the complexes with bt ligands exhibited more prominent charge-transfer (λ = 300 nm and above) (Tamayo et al., 2005). The peak maxima were somewhat similar to those of the corresponding [Ir(C^∧^N)_2_(Me-bpy-Me)]^+^ complexes in acetonitrile (Table 1).

**Table 1:** UV-Vis absorbance and luminescence peak maxima of the metal complexes at room temperature and low temperature.

**Complex**	**λ_abs_ (nm)**	**λ_em_ (r.t.)^*a*^/nm**	**λ_em_ (85 K)^*b*^/nm**
[Ir(bt)_2_(TEG-bpy-TEG)]Cl	209, 308, 321, 374b, 410^*c*^	531, 568, 619^*d*^	517, 557, 599
[Ir(bt)_2_(Me-bpy-TEG)]Cl	209, 308, 321, 374b, 410^*c*^	531, 567, 613^*d*^	
[Ir(bt)_2_(Me-bpy-Me)]PF_6_	256, 408^*c*^, 468^*c*^	528, 568, 615^*d*^	516, 558, 606
[Ir(ppy)_2_(TEG-bpy-TEG)]Cl	211s, 253, 268^*d*^, 311^*d*^, 376^*c*^, 410^*c*^	628	471, 511^*d*^, 533
[Ir(ppy)_2_(Me-bpy-TEG)]Cl	211s, 253, 268^*d*^, 311^*d*^, 376^*c*^, 410^*c*^	623	
[Ir(ppy)_2_(Me-bpy-Me)]PF_6_	208, 269, 321, 411^*c*^	590	473, 511, 533
[Ru(bpy)_3_]Cl_2_	215, 290, 445	629	581, 629, 685^*d*^

a *10 μM in water, except for the two [Ir(C^∧^N)_2_(Me-bpy-Me)]PF_6_ complexes, which were prepared at 10 μM in acetonitrile*.

b *5 μM in 4:1 (v/v) ethanol:methanol*.

c *broad*.

d *shoulder*.

**Figure 3:**
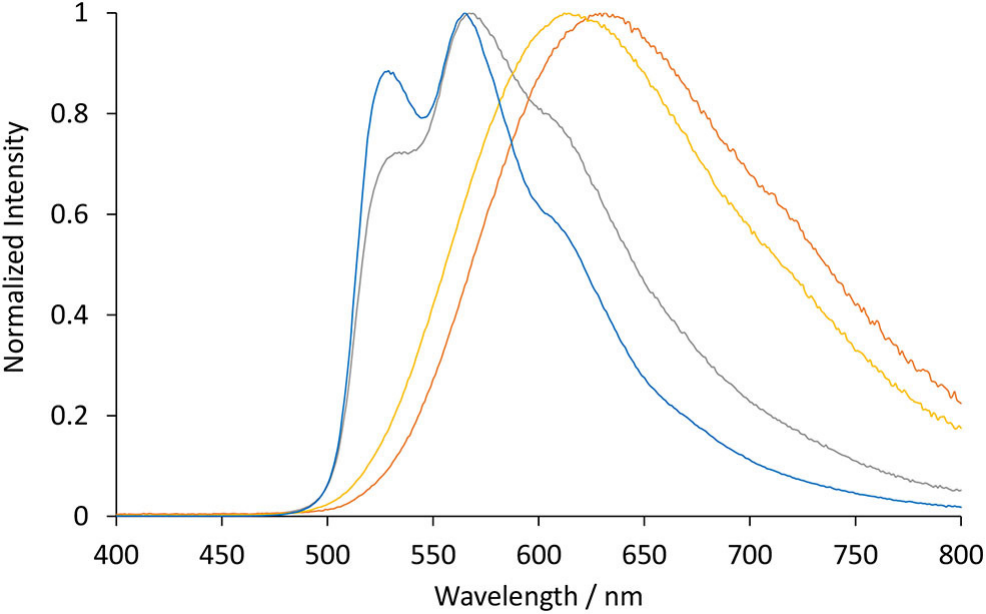
Normalized luminescence emission spectra obtained for [Ir(bt)_2_(TEG-bpy-TEG)]^+^ (gray line), [Ir(bt)_2_(Me-bpy-TEG)]^+^ (blue line), [Ir(ppy)_2_(TEG-bpy-TEG)]^+^ (orange line), and [Ir(ppy)_2_(Me-bpy-TEG)]^+^ (yellow line), at 10 μM in water at ambient temperature. The excitation wavelength was 308 nm for [Ir(bt)_2_(N^∧^N)]^+^ complexes, and 260 nm for [Ir(ppy)_2_(N^∧^N)]^+^ complexes. Spectra were corrected for the change in instrument sensitivity across the wavelength range with a correction factor established using a light source with standard spectral irradiance.

The photoluminescence spectra of the four novel complexes ([Ir(ppy)_2_(TEG-bpy-TEG)]Cl, [Ir(bt)_2_(TEG-bpy-TEG)]Cl, [Ir(ppy)_2_(Me-bpy-TEG)]Cl and [Ir(bt)_2_(Me-bpy-TEG)]Cl) and the archetype ECL metal complex [Ru(bpy)_3_]^2+^ were initially examined at room temperature at 10 μM in aqueous solution (Figure 3 and Table 1). The peak maxima were also compared to those of the Ir(ppy)_2_(Me-bpy-Me)]PF_6_ and [Ir(bt)_2_(Me-bpy-Me)]PF_6_ complexes at the same concentration in acetonitrile, which were in reasonable agreement with previously reported data (Lepeltier et al., 2005; Zanoni et al., 2014).

**Figure 4:**
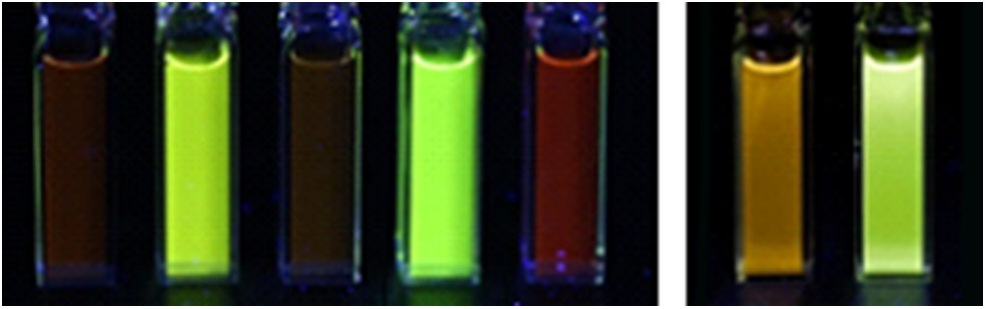
Photographs of luminescence under UV light. Left: [Ir(ppy)_2_(TEG-bpy-TEG)]Cl, [Ir(bt)_2_(TEG-bpy-TEG)]Cl, [Ir(ppy)_2_(Me-bpy-TEG)]Cl, [Ir(bt)_2_(Me-bpy-TEG)]Cl and [Ru(bpy)_3_]Cl_2_, in water at a concentration of 0.1 mM. Right: Ir(ppy)_2_(Me-bpy-Me)]PF_6_ and [Ir(bt)_2_(Me-bpy-Me)]PF_6_ at 0.1 mM in acetonitrile.

The three [Ir(bt)_2_(N^∧^N)]^+^ complexes exhibited similar peak maxima (Table 1). There were differences in the relatively intensity of the three major emission bands (Figure 3), but the overall emission colors of the two novel complexes in water and the Me-bpy-Me analog in acetonitrile were visually a similar green (Figure 4, second, fourth and seventh cuvette from the left). The luminescence of heteroleptic iridium(III) complexes has previously been attributed to mixed ligand-centered (^3^LC (π → π^*^) and metal-to-ligand charge-transfer ^3^MLCT [dπ(Ir) → π^*^(C^∧^N)] transitions (Lowry and Bernhard, 2006). The vibronic fine structure observed in the emission spectra of the bt complexes is consistent with a significant π → π^*^ contribution to the luminescence.

The two novel [Ir(ppy)_2_(N^∧^N)]^+^ complexes in water exhibited single broad emission peaks (Figure 3) characteristic of a ^3^MLCT excited state predominantly localized on the bpy (N^∧^N) ligand (Pomarico et al., 2016). The red-shift of the closely related [Ir(ppy)_2_(Me-bpy-Me)]^+^ by over 30 nm in acetonitrile relative to [Ir(ppy)_2_(Me-bpy-TEG)]^+^ and [Ir(ppy)_2_(TEG-bpy-TEG)]^+^ in water, which was not observed with the bt analogs (Table 1), is consistent with the solvochromic shifts previously reported for [Ir(ppy)_2_(bpy)]^+^ and [Ir(ppy)_2_(dtb-bpy)]^+^ at ambient temperature (Wu et al., 2010; Yen et al., 2016; Connell et al., 2019). Some aggregation of the complexes is possible, considering they possess a hydrophilic core with polar side chain(s), which may enhance this effect. The lower emission intensities of the [Ir(ppy)_2_(TEG-bpy-TEG)]^+^, [Ir(ppy)_2_(Me-bpy-TEG)]^+^, and [Ru(bpy)_3_]^2+^ complexes in water (Figure 4) compared to the [Ir(ppy)_2_(Me-bpy-Me)]^+^ in acetonitrile, under a UV lamp, are in part due to the lower sensitivity of the camera (and eye) at 623–629 nm compared to 590 nm. Quantitative comparisons of photoluminescence intensities or quantum yields were not undertaken as they are not well correlated with ECL intensities (Barbante et al., 2014a).

**Figure 5:**
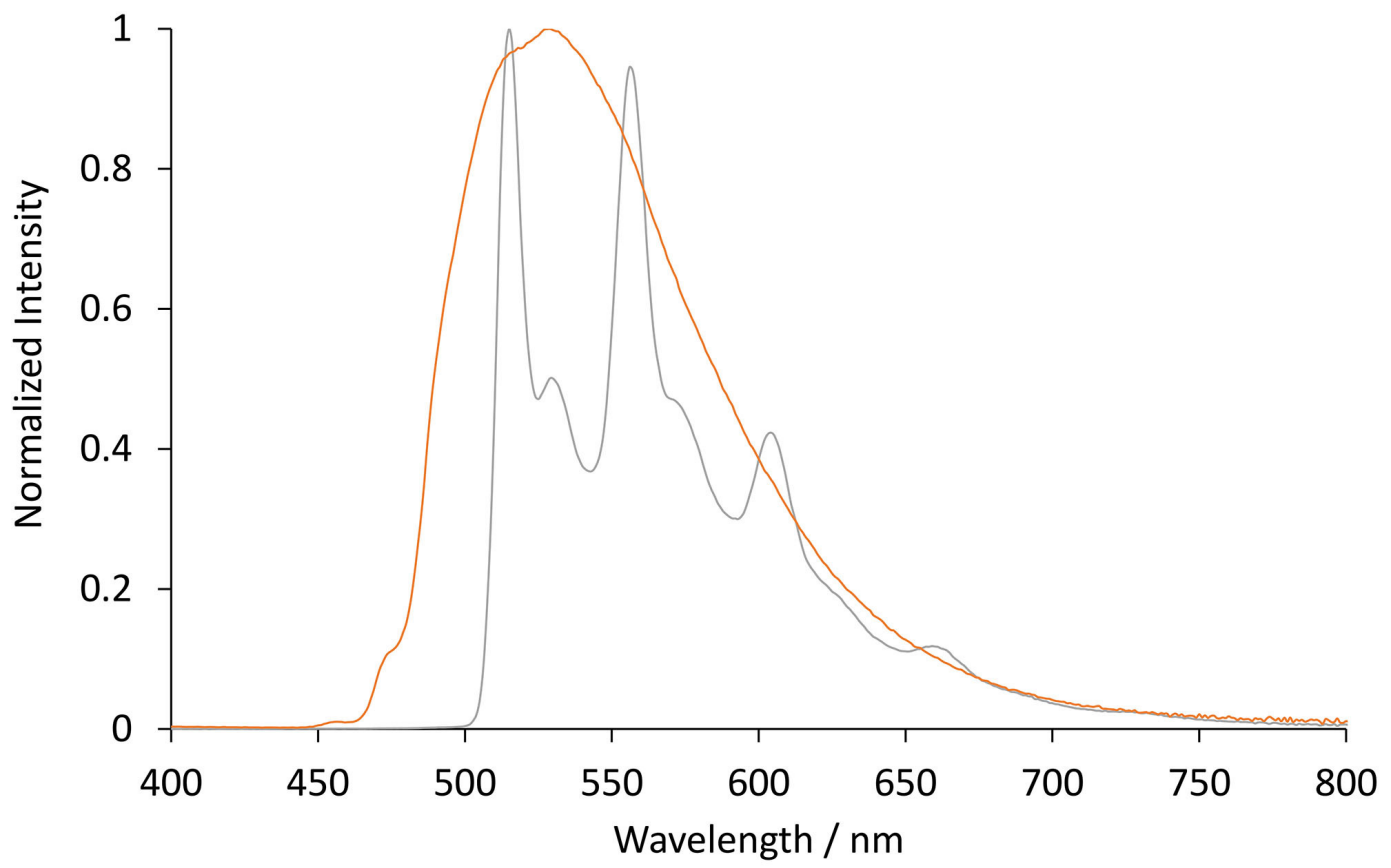
Normalized photoluminescence emission spectra obtained for [Ir(bt)_2_(TEG-bpy-TEG)]^+^ (gray line), and [Ir(ppy)_2_(TEG-bpy-TEG)]^+^ (orange line), at 5 μM in 4:1 (v/v) ethanol:methanol at low-temperature (85 K). Spectra were corrected for the change in instrument sensitivity across the wavelength range with a correction factor established using a light source with standard spectral irradiance.

The photoluminescence spectra of the two [Ir(C^∧^N)_2_(TEG-bpy-TEG)]^+^ complexes (where C^∧^N is bt or ppy) were examined in 4:1 (v/v) ethanol:methanol at 85 K (Figure 5). Low-temperature spectra generally show greater detail of vibrational energy levels, and allow for a more accurate estimation of the energy gap (*E*_00_) between the lowest vibrational levels of the ground and lowest excited state (Jones and Fox, 1994). The low temperature spectrum for [Ir(bt)_2_(TEG-bpy-TEG)]^+^ is highly structured (Figure 5), even more than at room temperature (Figure 3), and was in close agreement with that of [Ir(bt)_2_(Me-bpy-Me)]^+^ (Table 2). The highest energy peak at 517 nm corresponds to an *E*_00_ energy of 2.4 eV. The broad emission spectrum produced by [Ir(ppy)_2_(TEG-bpy-TEG)]^+^ at low temperature is unusual for an iridium(III) complex, and the highest energy band at 471 nm is well over 100 nm blue-shifted from that of the room-temperature spectrum. The analogous hypsochromic shift of the closely related [Ir(ppy)_2_(Me-bpy-Me)]^+^ complex has been ascribed to not only the rigidochromic phenomena typically observed with ^3^MLCT emissions, but also contribution from higher energy transitions due to an unusually high barrier for relaxation to the ^3^MLCT_(bpy)_ (King and Watts, 1987; Wu et al., 2010; Yen et al., 2016; Connell et al., 2019). Although this complicates the approximation of *E*_00_ for [Ir(ppy)_2_(TEG-bpy-TEG)]^+^, comparison with the interpretation of spectra of related complexes (Connell et al., 2019) enables an estimation at 2.4 eV.

**Table 2:** Reduction potentials of the complexes.

**Complex**	***E_*p*_*^ox^/V (vs. Ag/AgCl)*^*a*^***	***E^0^′*(M^+^/M) (V vs. Fc^+/0^)*^*b*^***	***E^0^′*(M/M^−^) (V vs. Fc^+/0^)^*b*^**
[Ir(bt)_2_(TEG-bpy-TEG)]Cl	1.25	1.02	−1.73
[Ir(bt)_2_(Me-bpy-TEG)]Cl	1.22	1.00	−1.79
[Ir(bt)_2_(Me-bpy-Me)]PF_6_	-	1.03	−1.79
[Ir(ppy)_2_(TEG-bpy-TEG)]Cl	0.84, 1.18	0.86	−1.79
[Ir(ppy)_2_(Me-bpy-TEG)]Cl	0.73, 1.04	0.86	−1.81
[Ir(ppy)_2_(Me-bpy-Me)]PF_6_	-	0.85	−1.86
[Ru(bpy)_3_](PF_6_)_2_	-	0.89	−1.73

a *1 mM in buffered aqueous solution*.

b *0.1 mM in acetonitrile with 0.1 M TBAPF_6_ electrolyte*.

### Voltammetry

**Figure 6:**
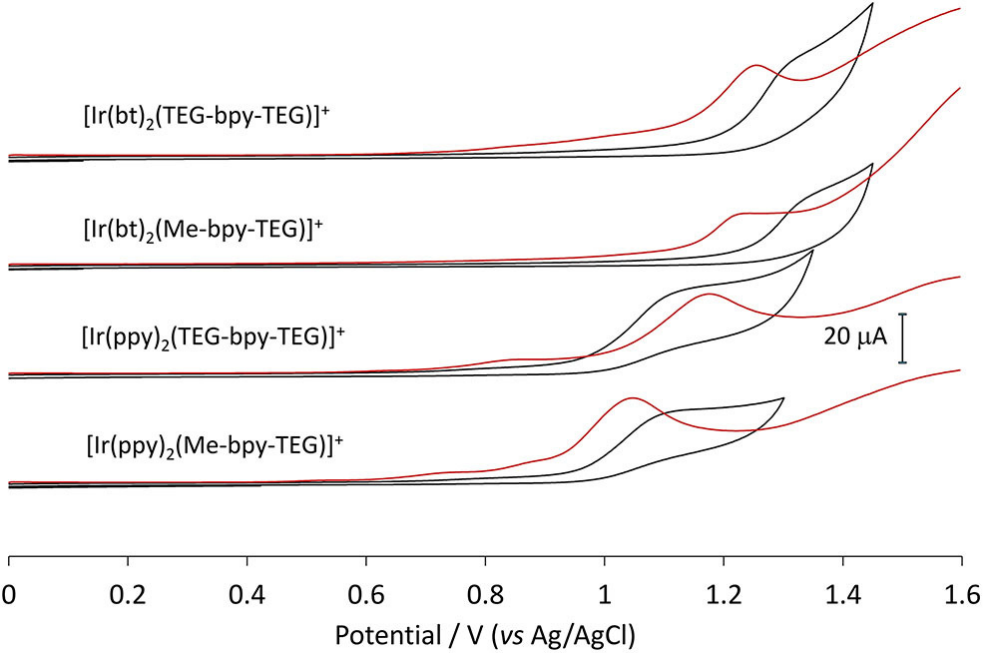
Squarewave and cyclic voltammetry traces obtained for the four novel iridium(III) complexes in buffered aqueous solution at a concentration of 1 mM.

Cyclic voltammetry (CV) experiments were initially conducted using buffered aqueous solutions to mimic the analytical conditions for which they were designed (Figure 6, black lines). The oxidation of the four novel complexes appears irreversible in an aqueous environment. The shape of the cyclic voltammograms made assigning peak potentials difficult, so squarewave voltammetry (Figure 6, orange lines) was also conducted to inform the positions of the oxidation peaks (Table 2).

**Figure 7:**
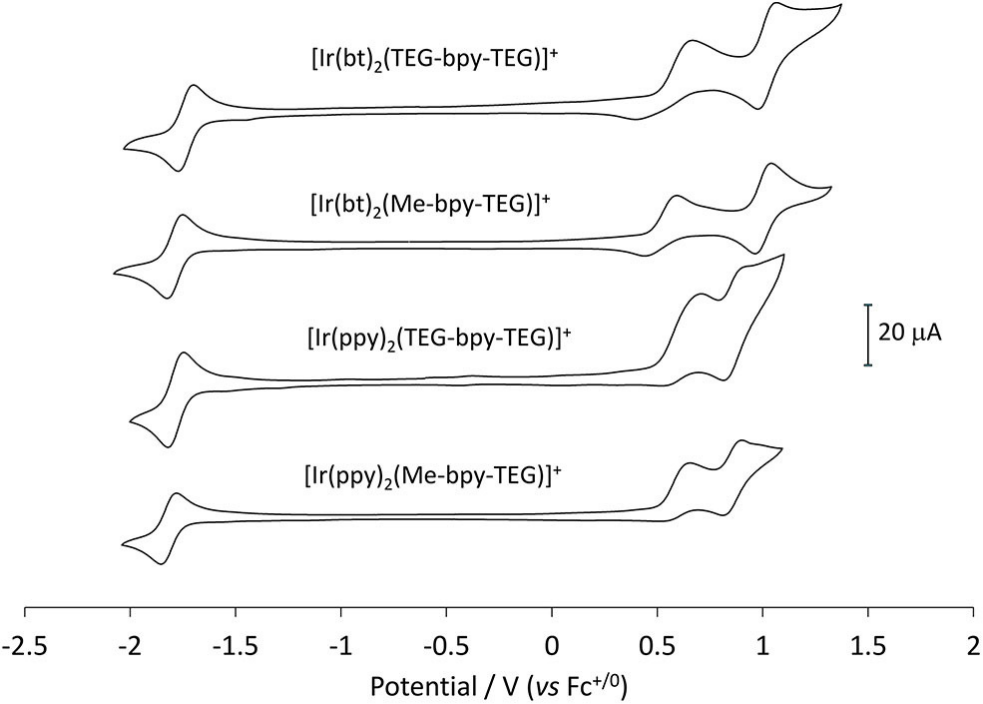
Cyclic voltammetry traces for the four novel iridium(III) complexes at 1 mM in acetonitrile with 0.1 M TBAPF_6_ electrolyte.

The mechanism of co-reactant ECL depends on both the oxidation and reduction of the metal complex, so it is important that both are characterized. The reduction of the complexes, however, is obscured in voltammetric experiments due to the reduction of solvent, so these potentials were determined in acetonitrile and referenced to the ferrocenium/ferrocene couple (Figure 7). This internal electrochemical reference is more reliable that the reference electrode potential and provides a more accurate comparison to the previously reported potentials of related iridium complexes that were not sufficiently soluble in an aqueous buffer (Table 2). The additional oxidation peak at ~0.6 V (vs. Fc^+/0^) in the traces in Figure 7 arises from the chloride counter ion of these complexes. This peak could be removed by converting the compounds to their hexafluorophosphate salts, but this was deemed unnecessary.

The values obtained for the three [Ir(bt)_2_(N^∧^N)]^+^ complexes were very similar (oxidation potentials within 30 mV and reduction potentials within 60 mV). Those obtained for the three [Ir(ppy)_2_(N^∧^N)]^+^ complexes were also consistent (oxidation potentials within 10 mV and reduction potentials within 70 mV). The similarity of these potentials indicates that the presence of TEG moieties on the bpy ligand has very little influence on the electrochemical properties of the complex.

### Electrogenerated Chemiluminescence

**Figure 8:**
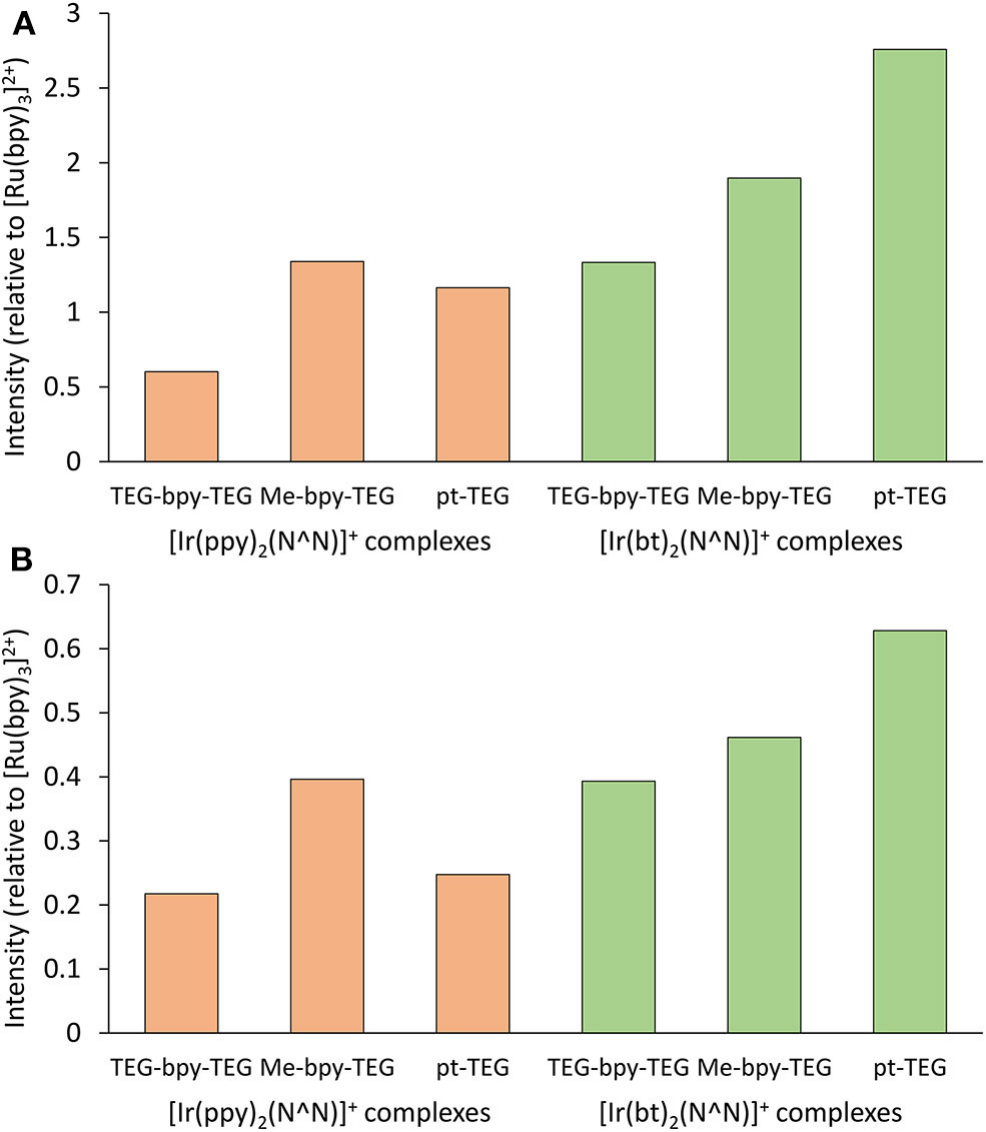
Relative ECL intensities (integrated area of ECL spectra obtained using a CCD spectrometer) of water-soluble [Ir(C^∧^N)_2_(N^∧^N)]^+^ complexes at 10 μM in a 0.1 M phosphate buffer with 10 mM TPrA, using an applied potential of $E_{\text{p}}^{\text{ox}}$ + 0.1 V for each complex for **(A)** 0.1 s or **(B)** 0.5 s. Experiments were performed in duplicate and the intensities were averaged.

The ECL intensities of the [Ir(C^∧^N)_2_(N^∧^N)]^+^ complexes containing a Me-bpy-TEG or TEG-bpy-TEG ligand in buffered aqueous solution using TPrA as a co-reactant were compared those of the analogous complexes with a pt-TEG ligand. To remove the bias in sensitivity of typical photomultiplier tubes toward the hypsochromic emissions of these complexes, we used the integrated area of ECL spectra collected using a CCD spectrometer for these comparisons. Figure 8 shows the ECL intensities relative to that of the [Ru(bpy)_3_]^2+^ complex under the same conditions.

The co-reactant ECL intensities of iridium(III) complexes relative to [Ru(bpy)_3_]^2+^ can be highly dependent on instrumental and chemical conditions (Chen et al., 2017). Using an applied potential pulse at [$E_{\text{p}}^{\text{ox}}$ + 0.1 V] for 0.1 s, the co-reactant ECL intensities of most of the [Ir(C^∧^N)_2_(N^∧^N)]^+^ complexes were greater than that of [Ru(bpy)_3_]^2+^ (Figure 8A), but when the pulse time was increased to 0.5 s (Figure 8B) the intensities were below that of [Ru(bpy)_3_]^2+^. Nevertheless, the trend in intensities between the [Ir(C^∧^N)_2_(N^∧^N)]^+^ complexes was similar at the two pulse times. The intensities of the [Ir(C^∧^N)_2_(Me-bpy-TEG)]^+^ complexes were between 1.2- and 2.2-fold those of the [Ir(C^∧^N)_2_(TEG-bpy-TEG)]^+^.

To apply the well-known mechanisms of ECL established for [Ru(bpy)_3_]^2+^ with TPrA co-reactant (Miao et al., 2002) to other metal complexes with the same co-reactant, their potentials and excited state energies need to be considered (Stringer et al., 2014; Kerr et al., 2016; Chen et al., 2019). Electrochemical oxidation of the TPrA co-reactant (*E*^0^ ≈ 0.5 V vs. Fc^+/0^) initially generates the corresponding aminium radical cation (CH_3_CH_2_CH_2_)_3_N^•+^, denoted TPrA^•+^, which deprotonates to form the strongly reducing α-aminoalkyl radical Pr_2_NC^•^HCH_2_CH_3_ (denoted TPrA^•^, *E*^0^ ≈ −2.1 V vs. Fc^+/0^), shown below as reactions 1 and 2 (Smith and Mann, 1969; Noffsinger and Danielson, 1987; Leland and Powell, 1990; Miao et al., 2002).

(1) $$\begin{array}{r} \text{TPrA}-{\text{e}}^{-}\to \text{TPr}{\text{A}}^{•+} \end{array}$$

(2) $$\begin{array}{r} \text{TPr}{\text{A}}^{•+}\to \text{TPr}{\text{A}}^{•}+{\text{H}}^{+} \end{array}$$

If the metal complex is also oxidized (reaction 3, where M = [Ru(bpy)_3_]^2+^ or [Ir(C^∧^N)_2_(N^∧^N)]^+^), subsequent reaction with the α-aminoalkyl radical may generate ECL (reactions 4 and 5) if there is sufficiently energy to attain the excited state, which can be estimated using: *E*°(M^+^/M)–*E*°(TPrA^•^) > *E*_00_(M)). If *E*°(M^+^/M) > *E*°(TPrA), the co-reactant can also be oxidized *via* the ‘catalytic' route shown in reaction 6.

(3) $$\begin{array}{r} \text{M}-e^{-}\to {\text{M}}^{+} \end{array}$$

(4) $$\begin{array}{r} {\text{M}}^{+}+\text{TPr}{\text{A}}^{•}\to {\text{M}}^{*}+\text{otherproducts} \end{array}$$

(5) $$\begin{array}{r} {\text{M}}^{*}\to \text{M}+h\nu \end{array}$$

(6) $$\begin{array}{r} {\text{M}}^{+}+\text{TPrA}\to \text{M}+\text{TPr}{\text{A}}^{+•} \end{array}$$

Moreover, the TPrA^•^ radical may reduce the metal complex (reaction 7, feasible if *E*°(M/M^−^) > *E*°(TPrA^•^)), and the electronically excited state can be attained *via* the annihilation pathway (reaction 8, provided *E*°(M^+^/M)–*E*°(M/M^−^) > *E*_00_(M)).

(7) $$\begin{array}{r} \text{M}+\text{TPr}{\text{A}}^{•}\to {\text{M}}^{-}+\text{otherproducts} \end{array}$$

(8) $$\begin{array}{r} {\text{M}}^{+}+{\text{M}}^{-}\to {\text{M}}^{*}+\text{M} \end{array}$$

Based on the data presented above, reactions 1–8 are feasible for these iridium(III) complexes. An alternative pathway to the excited state, important for bead-based assays in which the majority of the metal complex luminophores cannot be electrochemically oxidized (Miao et al., 2002; Chen et al., 2019), involves reactions 1, 2, 7, and 9, where the reduced metal complex reacts with the aminium radical cation. This pathway requires not only *E*°(M/M^−^) > *E*°(TPrA^•^), but also *E*°(TPrA^•^)–*E*°(M/M^−^) > *E*_00_(M).

(9) $$\begin{array}{r} {\text{M}}^{-}+\text{TPr}{\text{A}}^{+•}\to {\text{M}}^{*}+\text{TPrA} \end{array}$$

Despite the similarity of their *E*°(M/M^−^), the iridium(III) complexes exhibit higher excited state energies than [Ru(bpy)_3_]^2+^. Considering the requirements noted above, the data presented in Tables 1, 2 suggest that reaction 9 will be slightly energy insufficient (~0.1–0.2 V), limiting the application of these electrochemiluminophores to assays in which the metal complex can be oxidized. These calculations, however, involve considerable error, including those associated with estimating *E*°(TPrA), *E*°(TPrA^•^) (Miao et al., 2002) and *E*_00_(M) (Jones and Fox, 1994; Connell et al., 2019), and the use of potentials measured in acetonitrile for a ECL reaction in aqueous solution. Further investigations are needed to confirm the ECL pathways for these novel luminophores.

**Figure 9:**
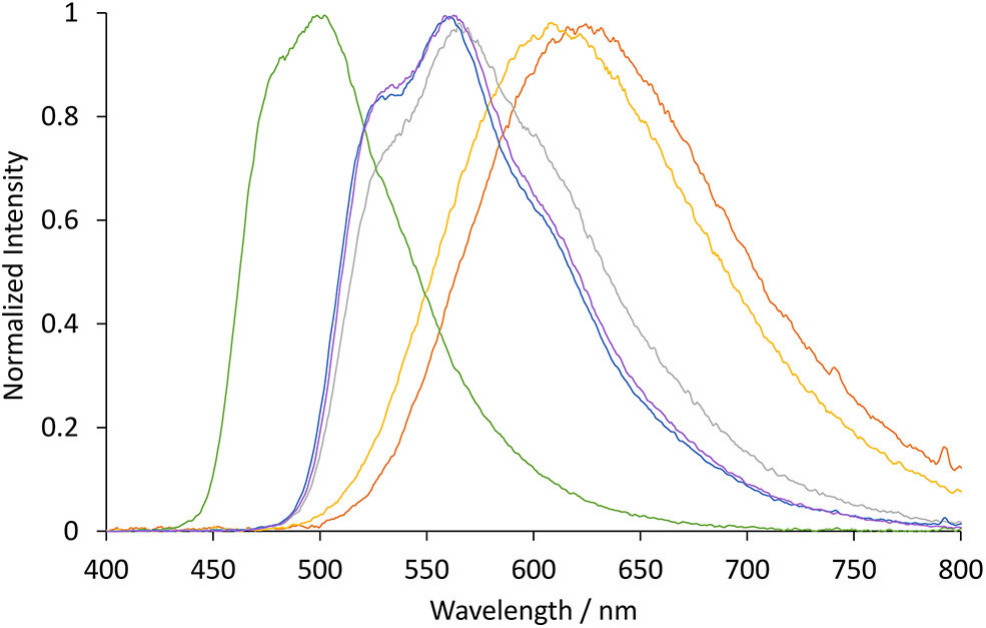
Normalized ECL spectra obtained for [Ir(bt)_2_(TEG-bpy-TEG)]^+^ (gray line), [Ir(bt)_2_(Me-bpy-TEG)]^+^ (blue line), [Ir(bt)_2_(pt-TEG)]^+^ (purple line), [Ir(ppy)_2_(TEG-bpy-TEG)]^+^ (orange line), [Ir(ppy)_2_(Me-bpy-TEG)]^+^ (yellow line) and [Ir(ppy)_2_(pt-TEG)]^+^ (green line) at 10 μM in 0.1 M phosphate buffer with 10 mM TPrA co-reactant, using an applied potential of *E*_*p*_^ox^ + 0.1 V for each complex for 0.1 s.

The ECL spectra of [Ir(C^∧^N)_2_(Me-bpy-TEG)]^+^ and [Ir(C^∧^N)_2_(TEG-bpy-TEG)]^+^ (Figure 9) were in good agreement with their photoluminescence emission spectra (Figure 3), taking into account the lower resolution of the CCD spectrometer used to collect the ECL spectra. The spectral distribution of [Ir(bt)_2_(pt-TEG)]^+^ is similar to that of [Ir(bt)_2_(Me-bpy-TEG)]^+^ and [Ir(bt)_2_(TEG-bpy-TEG)]^+^, but emission of [Ir(ppy)_2_(pt-TEG)]^+^ is considerably blue-shifted from [Ir(ppy)_2_(Me-bpy-TEG)]^+^ and [Ir(ppy)_2_(TEG-bpy-TEG)]^+^ (Figure 9). A practical outcome of this shift is that much greater ECL intensities will be measured with [Ir(ppy)_2_(pt-TEG)]^+^ than with [Ir(ppy)_2_(Me-bpy-TEG)]^+^ or [Ir(ppy)_2_(TEG-bpy-TEG)]^+^ when using photomultiplier tubes that are much more sensitive toward shorter wavelengths of light within the visible region. On the other hand, as both the Me-bpy-TEG and pt-TEG ligands can be readily adapted for bioconjugation, this shows a viable strategy to create two ECL-labels with distinctly different emission colors from the same commercial [Ir(ppy)_2_(μ-Cl)]_2_ dimer that provide similar ECL intensities using a CCD spectrometer to distinguish their emissions. The novel [Ir(bt)_2_(TEG-bpy-TEG)]^+^ and [Ir(ppy)_2_(TEG-bpy-TEG)]^+^ complexes also exhibit distinct spectral distributions. As noted in previous studies (Doeven et al., 2012; Cao et al., 2020), the number of luminophores in multi-color ECL may be limited by the broad emission spectra of the metal complexes. The difference in their oxidation potentials (Table 1), however, may also enable “potential-resolved” ECL, which has recently been exploited to expand the scope of multi-color systems (Doeven et al., 2015b; Guo et al., 2018; Moghaddam et al., 2019; Bouffier and Sojic, 2020).

## Conclusions

Four [Ir(C^∧^N)_2_(N^∧^N)]Cl complexes in which C^∧^N = ppy or bt, and N^∧^N = bpy with either one or two TEG groups attached in the 4 and 4′ positions, were successfully synthesized with acceptable yields for all reaction steps. Characterization of the complexes showed that the introduction of one or two the TEG groups to the bpy ligand of iridium(III) complexes is a useful strategy to enhance their solubility in aqueous solution while retaining the electrochemical and spectroscopic properties of the parent luminophore. The TEG groups also provide a convenient attachment point for the future development of ECL labels.

## Data Availability Statement

The raw data supporting the conclusions of this article will be made available by the authors, without undue reservation.

## Author Contributions

PF and DH conceived and designed the study. BN synthesized the compounds under the guidance of DH and LH. LC and BN performed the spectroscopic and electrochemical characterizations under the guidance of ED and PF. All authors contributed to manuscript preparation and revision and have read and approved the submitted version.

## Conflict of Interest

The authors declare that the research was conducted in the absence of any commercial or financial relationships that could be construed as a potential conflict of interest. The handling editor declared a past co-authorship with the authors DH and PF.
